# Cell-Free Fetal DNA for Prenatal Screening of Aneuploidies and Autosomal Trisomies: A Systematic Review

**DOI:** 10.1155/2024/3037937

**Published:** 2024-10-23

**Authors:** Kenza Benchekroun Belabbes, Elena Bendala Tufanisco, Chirag C. Sheth

**Affiliations:** ^1^Department of Medicine and Surgery, Faculty of Health Sciences, Universidad CEU Cardenal Herrera, CEU Universities, Valencia, Spain; ^2^Department of Biomedical Sciences, Faculty of Health Sciences, Universidad CEU Cardenal Herrera, CEU Universities, Valencia, Spain

**Keywords:** aneuploidy, cell-free fetal DNA, noninvasive prenatal testing, trisomy

## Abstract

**Aim:** This study was aimed at comparing the positive predictive value of a high-risk cell-free fetal DNA test result for sex chromosome aneuploidies (45,X0, 47,XXX, 47,XXY, and 47,XYY) and autosomal trisomies (T21, T18, and T13) with confirmatory tests in singleton pregnancies. Additionally, we identify the main reason for discordant and inconclusive results.

**Methods:** PubMed, Web of Science, and Scopus were searched from 2017 for primary research articles on cell-free fetal DNA testing of autosomal trisomies and sex chromosome aneuploidies in singleton pregnancies. The methodological characteristics and the statistical results of the studies were collected, and the risk of bias was assessed.

**Results:** Fourteen studies were included. Among the autosomal trisomies, T21 had the highest, whereas T13 showed the lowest positive predictive values. As for the sex chromosome aneuploidies, the lowest values were found with 45,X0. Although discordant and inconclusive results were reported inconsistently, false positives were mainly caused by mosaicism, and inconclusive results were mostly secondary to a low fetal DNA fraction.

**Conclusion:** Cell-free fetal DNA is a reliable screening tool for autosomal trisomies. It is also useful for sex chromosome aneuploidies, although the positive predictive values are lower. A positive screening result should be followed with a confirmatory test.

Summary

• There is extensive evidence supporting the use of cell-free fetal DNA for prenatal screening of autosomal trisomies (T21, T18, and T13) and sex chromosome aneuploidies, in singleton pregnancies.
• However, its accuracy needs to be greatly improved for sex chromosome aneuploidies, especially for 45,X0.
• Moreover, as it is a screening test and not a diagnostic test, all positive cffDNA results should be followed by a confirmatory procedure.

## 1. Introduction

Congenital anomalies comprise a wide range of abnormalities that are present at birth and are of prenatal origin [1]. Chromosomal anomalies are a subtype of congenital anomalies and may be classified as numerical (aneuploidies: trisomy and monosomy) or structural (translocation, deletion, and duplication) [2]. In turn, numerical chromosomal anomalies, which are the object of this systematic review, comprise autosomal aneuploidies and SCAs. The most common autosomal aneuploidy is Trisomy 21 (T21, Down syndrome), followed by Trisomy 18 (T18, Edwards syndrome) and Trisomy 13 (T13, Patau syndrome) [3]. SCAs include monosomies and trisomies: Turner syndrome (monosomy X, 45,X0), Klinefelter syndrome (47,XXY), triple X syndrome (47,XXX), and Jacobs syndrome (47,XYY) [4]. Prenatal screening protocols include analysis of autosomal trisomies and SCAs [5].

Cell-free fetal DNA (cffDNA) was first discovered by Lo et al. in 1997 [6], described as the percentage of cell-free DNA in the mother's peripheral blood of fetal origin (cffDNA is eliminated rapidly after delivery) [7]. It is used as a test for aneuploidies in noninvasive prenatal testing (NIPT) [8]. In addition to prenatal screening, cffDNA is also used for fetal sex determination, fetal Rhesus D genotyping, early identification of preeclampsia, fetal growth restriction prediction, and diagnosis of diseases such as cystic fibrosis and achondroplasia [7, 9, 10].

CffDNA testing has the advantage of being a noninvasive procedure, avoiding the risk of miscarriage associated with invasive prenatal testing [11]. It has recently generated interest, as it has the potential of reducing the rate of invasive prenatal testing of chromosomal anomalies [12], and as a consequence, a large number of studies have been published on this topic in recent years. Despite the existence of several systematic reviews, none have compared the accuracy of cffDNA testing for autosomal Trisomies 21, 13, and 18 and for SCAs, since 2017 [13]. The objective of this review is to investigate the predictive performance of cffDNA testing for prenatal screening of SCAs and of the common autosomal trisomies, compared with gold-standard confirmatory methods. The study is aimed at being of value to clinicians by constructing an up-to-date review of the available evidence on the topic.

## 2. Materials and Methods

This systematic review was conducted following the PRISMA guidelines.

### 2.1. Search Strategy

PubMed, Web of Science, and Scopus were the databases searched. The keywords used were “cell-free fetal DNA”, “non-invasive prenatal testing”, “aneuploidy”, and “trisomy”. An advanced search was conducted, combining keywords, synonyms, MeSH equivalents, Boolean Operators (“AND” and “OR”), and type of study (only primary research articles were accepted). The full search strategy for each of the databases is available in Supporting Information 4: Appendix S1. The only filter applied was the publication date (2017 or later, after the last best review). The final search was conducted on 18/09/2023.

### 2.2. Study Selection and Eligibility Criteria

Firstly, duplicate articles were eliminated from the bulk retrieval generated by the search. Next, articles were screened by title and abstract. The remaining articles were fully reviewed and assessed according to the eligibility criteria.

The eligibility criteria were as follows. Only primary research articles were included. Studies including pregnant women with a singleton pregnancy, who underwent a NIPT by cffDNA, were considered eligible. Furthermore, the cffDNA results must have been compared to confirmatory prenatal or postnatal tests. The abnormalities included were the most common autosomal trisomies, as described above, and SCAs.

Studies including diseases other than those previously stated were excluded. Multiple pregnancies (including vanishing twins) were also excluded, as they are commonly associated with inconclusive cffDNA results [14].

### 2.3. Data Extraction

Data was collected manually from the eligible studies, following the screening process.

The methodological characteristics of the studies were extracted: primary author, publication date, type of study, country, duration of study, number of participants, participants characteristics (maternal and gestational ages), cffDNA sequencing technology used, and diagnostic confirmation technique. Supporting Information 2: Table S1 summarizes these characteristics.

The statistical results comparing cffDNA to the confirmed (gold-standard) diagnoses of each study were collected for each aneuploidy: T21, T18, T13, and SCAs (45,X0, 47,XXY, 47,XXX, and 47,XYY). The positive predictive value (PPV) is presented as the key outcome variable in this systematic review as it was the common metric across the 14 studies, presented in Table 1 (for autosomal trisomies) and Table 2 (for SCAs). The prevalence of a positive NIPT test outcome was also collected for each of the studies (see Table 3 for autosomal trisomies and Table 4 for SCAs). The rate of inconclusive results was also collected.

When the overall PPV of several aneuploidies was given, the individual PPV for each aneuploidy was calculated when the data was available. As for the high-risk NIPT result prevalence, it was either directly provided or calculated as a percentage by dividing the number of high-risk (positive) cffDNA test results by the total number of samples tested.

### 2.4. Quality Assessment and Risk of Bias

The study selection, quality assessment, and data extraction were carried out following the PRISMA guidelines. The Critical Appraisal Skills Programme (CASP) checklists were used to assess the risk of bias [29].

## 3. Results

### 3.1. Search Results

A total of 1209 articles were retrieved from the initial database searches, and 512 duplicates were eliminated. Following the screening of titles/abstracts, 624 publications were excluded and 61 articles were retrieved for full review and eligibility assessment. Finally, 14 articles were considered eligible for inclusion (see the PRISMA flowchart; Supporting Information 1: Figure S1).

### 3.2. Included Study Characteristics

Supporting Information 2: Table S1 summarizes the methodological characteristics of the included studies. Of the 14 studies included in this review, 13 were retrospective [15–21, 23–28] and 1 was prospective [22]. Of the 13 retrospective studies, 12 consisted of observational or retrospective cohort studies [15, 16, 18–21, 23–28], and 1 was a nested case-control study [17]. The cohort size of the studies ranged from 200 to 93,048. The maternal age was unknown in 2 studies [19, 24], and the interquartile range was the only information given in one study [27]. Ten studies used massive parallel sequencing (MPS) technology as the DNA sequencing method [15–17, 19, 21–23, 25, 26, 28]. All the positive NIPT screening cases were supported by prenatal or postnatal karyotyping, provided that the participants consented to the procedure, and they were not lost to follow-up. In 4 studies [16, 18, 20, 21], both autosomal trisomies and SCAs were studied, whereas only autosomal trisomies were studied in 4 articles [15, 17, 19, 22], and only SCAs in 6 articles [23–28]. All in all, 8 studies for autosomal trisomies and 10 for SCAs were gathered.

### 3.3. Methodological Quality of Included Studies

The CASP tool was used to assess the risk of bias. A summary of the results is presented in Supporting Information 3: Table S2 and described in Supporting Information 5: Appendix S2.

### 3.4. Analysis of Autosomal Trisomies: 21, 18, and 13

Out of the 14 included studies, 8 studies evaluated the autosomal Trisomies 21, 18, and 13. The results are summarized in Table 1.

The cohort sizes range between 200 and 40311 participants (Supporting Information 2: Table S1) [21, 22]. Of the 8 studies, the highest prevalence of a positive cffDNA test result found for T21 was of 5.5% (Table 3) [17].

In general, the studies concurred in that T21 was found to have the highest PPVs as compared to T18 and T13 (Table 1). Three studies showed a 100% PPV for T21 [18, 19, 22]. Another two studies reported PPVs between 90% and 99% [16, 17] while PPVs of 80% to 90% were seen in two studies [20, 21].

The lowest relative overall PPVs were reported for T13. A PPV of 0%, followed by 14.3%, was the lowest value reported across the eight studies [19, 21]. Results were quite heterogenous, with PPVs as high as 100% [16, 17, 20], and a number of results between 20% and 90% was also reported [20, 21, 24]. One study reported no cases of Trisomy 13 and consequently did not present its PPV [18].

For T18, the results were intermediate between those of T21 and T13. Lee et al. and Serapinas et al. reported PPVs of 100% [17, 18]. Alyafee et al. found an 80% PPV [22]. The remaining four studies had results below 60%: 59%, 58.7%, 48.2%, and 0% [16, 19–21].

Among the eight studies that reported on the autosomal trisomies, one study omitted the individual PPVs and only included the overall PPV of the three trisomies, with a result of 64.7% [15]. However, a PPV of 100% was reported when the cases with multiple clinical indications, rather than just one, and including abnormal ultrasound findings for NIPT, were exclusively accounted for [15].

### 3.5. Analysis of Sex Chromosome Aneuploidies

Out of the 14 included studies, 10 studies involved analysis of SCAs. The results are summarized in Table 2.

The number of participants per study ranged from 862 to 93,048 (Supporting Information 2: Table S1) [18, 27]. The prevalence of a high-risk NIPT is presented individually for each SCA in six studies [18, 23–26, 28], while only the overall PPV for all SCAs is available for four studies [16, 20, 21, 27] (Table 4). Of all these studies, the highest prevalence of a positive cffDNA test is 0.6% [21].

In comparison to the other SCAs, the lowest PPVs were associated with Turner syndrome (all PPVs were < 30%) [23–28]. The PPVs for Klinefelter syndrome ranged from 25% to 77.8% [23–28]. In the case of triple X syndrome, four studies reported PPVs from 29.7% to 51.7% [25–28], while two studies reported a PPV of 100% [23, 24]. Finally, the studies which screened for Jacob's syndrome demonstrated heterogenous results. Two studies returned PPVs of 100% [18, 23], with others in the range of 60%–83.3% [24–27]. One study returned a low PPV of 27.3% [28]. It is pertinent to note that of the two studies reporting a PPV of 100%, the latter study only identified one case that bore the 47,XYY SCA.

Among the 10 studies that reported on SCAs, three studies reported only the overall PPV, without specifying the PPV of each individual SCA. They all presented PPVs < 50% [16, 20, 21].

### 3.6. Analysis of Discordant (False Positive (FP) and False Negative (FN)) Result

The incidence of FP and FN cases was not uniformly reported across the 14 studies.

Although the number of FP cases was documented in 13 of the studies, the cause of the result was only confirmed in four studies, while the others offered a generic (nonspecific) explanation. Ma et al. proved that a case wrongly screened as XYY was later confirmed as a microdeletion [24]. Placental mosaicism was a cause for one T18 FP case, while one T13 FP case was confirmed postnatally by placental investigation and karyotyping of cord blood, which explains the 0% PPVs found in this study for T18 and T13 [19]. Furthermore, two maternal mosaicism cases were confirmed by maternal karyotyping in a 2021 study [26]. Two 47,XXX FP cases were identified, secondary to undiagnosed maternal 47,XXX SCA, as confirmed by maternal blood karyotype [25]. The final study presented PPVs of 100% across all the identified aneuploidies, and therefore, no FP was detected [18].

In general, FN was poorly reported or not reported at all in most of the studies. A small number of studies proffered some basic explanation (without differentiating between FP and FN), while others specifically described the FN outcomes of their studies. Our analysis shows that no FN was identified in five studies. However, placental mosaicism was confirmed as a source of FN in a single study, in which three cases of T18 were identified by CVS with quantitative fluorescent polymerase chain reaction [17].

### 3.7. Inconclusive Results

Reporting of the percentage of no-call (or inconclusive) results was absent in 5 studies. Nevertheless, 10 of the 14 studies stated that the main cause for no-call results was an insufficient cffDNA fraction (defined as a fetal fraction of < 4% in 9 studies). In one study, it was defined as < 2.8%, and the median fetal fraction in the no-call group was significantly lower (3.1%) than that in the group with a conclusive result (9.1%) [18].

In addition to an insufficient cffDNA fraction, other reasons cited for the no-call results were as follows: unusually high total free DNA, failure to pass the quality control measures, and sequencing run failure [15, 25, 28].

In most cases, the sample was successfully rerun, giving a valid result and a much lower final no-call rate compared to that of the first sample run. In one study, 94% of the no-call results were successfully rerun with the same sample, whereas three cases required a third rerun to obtain results [17]. Similarly, there were originally 509 samples with a no-call result in a 2020 study, which was reduced to 46 samples after retesting [21]. As for the 2021 study by Luo t al., 38 participants had a definitive no-call, as opposed to 468 participants after the first sample run [28].

## 4. Discussion

### 4.1. Main Findings

The results have demonstrated that NIPT with cffDNA is reliable in the screening of autosomal trisomies. Overall, T21 had the most promising results, whereas T13 had the least promising. NIPT is also useful for SCAs screening, although it has yielded poorer results overall than those for the autosomal trisomies. Further work is required to improve PPV of SCAs, especially for 45,X0. All study authors agreed that confirmation by invasive procedures is necessary in all cases of high-risk cffDNA results.

### 4.2. Interpretation of the Results

#### 4.2.1. Aneuploidies

Among the autosomal trisomies, T21 had the highest and T13 had the lowest PPVs. Among the SCAs, the lowest PPVs were found for 45,X0. For each aneuploidy, the results oscillated greatly between the studies.

It is important to highlight that the patients with high-risk NIPT results who declined further invasive testing could have significantly impacted the resulting PPV, either increasing it if they were true positives or decreasing it if they were false positives.

#### 4.2.2. Discordant Results

Most of the studies only listed some general causes of false results. Mosaicism can be of maternal or fetal origin, given that as maternal age increases, a natural loss of the X chromosome results in a maternal cell-free DNA with less X fragments and that for SCAs, many cases can have multiple cell lines [22, 24, 27]. Another vastly mentioned cause of discordant results is the presence of a vanishing twin, which justifies classifying this factor as an exclusion criteria in our study.

A number of studies confirmed the origin of the discordant results: microdeletion, undiagnosed maternal SCA, and mosaicism. These are all well-known causes and are in accordance with a prior systematic review, which found mosaicism to be among the main reasons of discordant results [30].

Although the causes of FP were not verified in most studies, as they would require further testing, such as placental investigation, the number of FP cases was detailed, as this value is needed to calculate the PPV.

With regard to FN, they were poorly reported or not reported at all. No FNs were identified in five studies. However, the underestimation of FNs is very likely given that confirming FNs would require a karyotype of the fetus/newborn, following a negative cffDNA test. A phenotypical postnatal follow-up would not be sufficient to discard an aneuploidy, especially for SCAs as they are phenotypically diverse and can be undetectable at birth [23, 28]. In fact, historically, 75%–90% of patients with SCAs remain undiagnosed in their lifetime [31].

#### 4.2.3. Inconclusive Results

The main cause for no-call results was an insufficient cffDNA fraction. The cutoff value was different in one study due to the different DNA sequencing techniques used (single nucleotide polymorphism technology) (see Supporting Information 2: Table S1), which is claimed to provide accurate results with fetal fractions ≥ 2.8% (as opposed to ≥ 4% by MPS), and yet, the median fraction was of 3.1% in the no-call group [18].

The no-call results were missing in five studies, possibly because an inconclusive result could have been immediately discarded or successfully automatically rerun. In one study, all the fetal fractions were ≥ 4% (4%–31%), which may explain the absence of no-call results [22]. Consequently, the fetal fraction significantly impacts cffDNA testing.

While in most cases, the sample was successfully rerun, a second blood draw was sometimes necessary, contrary to the recommendation of the American College for Medical Genetics and Genomics [32].

#### 4.2.4. Interpretation of the Results in the Context of Other Evidence

Over the years, although various systematic reviews assessing the usefulness of cffDNA in prenatal screening have been published, not many focused on the value of cffDNA testing for autosomal trisomies and for SCAs and on their comparison. In concordance with our results, a 2017 systematic review and bivariate meta-analysis concluded that NIPT by cffDNA is an accurate tool in the screening of autosomal trisomies and 45,X0 and that FN and inconclusive results were poorly reported [13]. A 2017 meta-analysis on cffDNA demonstrated that cffDNA screening in singleton pregnancies could detect > 99% of fetuses with T21, 98% of those with T18, and 99% of those with T13; however, the number of reported SCA cases was too low to be accurately assessed [33]. Also, consistent with our findings, a recent systematic review found that NIPT is a reliable screening test for SCAs [34].

### 4.3. Strengths and Limitations

This systematic review was conducted according to the PRISMA 2020 guidelines. A thorough review of the available articles related to the topic was performed on three databases.

Compared with a 2017 systematic review and meta-analysis, in which prior studies were included, other factors related to cffDNA were investigated and a meta-analysis of the results was conducted. However, the PPV, a relevant indicator of accuracy, was not reported [13]. Furthermore, of all the SCAs, only 45,X0 was investigated [13]. The reason for the fewer studies in our systematic review is due to the focus on aneuploidies, excluding all the other uses of cffDNA.

This review has some limitations. Firstly, PPV was the only metric used and therefore presents its own biases. Secondly, more larger scale studies are needed. Thirdly, given that pregnancy outcome was not an objective of this review, this aspect was not considered. In addition, the differences in the DNA sequencing techniques used in each study were not considered. Finally, there were publications that were not retrieved, despite our best attempts to contact the authors.

### 4.4. Implications for Clinical Practice

The findings of this review cautiously support the use of cffDNA testing as a screening test for T21, T18, T13, 45,X0, 47,XXY, 47,XXX, and 47,XYY. However, despite the high PPV, we do not recommend its use as a diagnostic test, and this should be followed by an invasive confirmatory diagnostic technique.

## 5. Conclusion

The findings of this systematic review demonstrate that there is extensive evidence supporting the use of cffDNA for prenatal screening of the common autosomal trisomies (T21, T18, and T13) and SCAs, in singleton pregnancies. Among the autosomal trisomies, T21 had the highest results, whereas T13 had the lowest ones. As for SCAs, its accuracy needs to be greatly improved, especially for 45,X0. However, it is a screening test and not a diagnostic test; hence, all positive cffDNA results should be followed by a confirmatory procedure, as false positives can occur. Similarly, a negative result does not discard an aneuploidy, as false negatives can also occur. Finally, the main reason for false results is placental mosaicism, and the main cause of no-call results is an insufficient fraction of cffDNA.

### 5.1. Implications for Future Research

New studies with long-term follow-up or postnatal karyotyping for all cases (high-risk and low-risk NIPT results) are needed, especially for SCAs. Systematic reviews in the following populations are crucial to understand the usefulness of cffDNA screening in all types of patients: multiple pregnancies, rare autosomal trisomies, and other chromosomal abnormalities. The implementation of studies and systematic reviews on the other uses of cffDNA, such as vascular complications in pregnancy, could be of great interest.

## Figures and Tables

**Table 1:** Positive predictive values of autosomal trisomies.

	**T21 PPVs**	**T13 PPVs**	**T18 PPVs**
Taneja et al. (2017) [15]	Overall: 64.7%		
Liang et al. (2018) [16]	92%	23.1%	59%
Lee et al. (2019) [17]	98.3%	90%	100%
Serapinas et al. (2020) [18]	100%	0 cases	100%
Wan et al. (2020) [19]	100%	0%	0%
Lu et al. (2020) [20]	84.7%	42%	58.7%
Luo et al. (2020) [21]	84%	14.3%	48.2%
Alyafee et al. (2021) [22]	100%	100%	80%

Abbreviations: PPV: positive predictive value; T13: trisomy 13; T18: trisomy 18; T21: trisomy 21.

**Table 2:** Positive predictive values of SCAs.

	**45,X0 PPVs**	**47,XXY PPVs**	**47,XXX PPVs**	**47,XYY PPVs**
Zhang et al. (2017) [23]	29.4%	77.8%	100%	100%
Liang et al. (2018) [16]	Overall: 49.1%			
Ma et al. (2018) [24]	22.2%	25%	100%	60%
Deng et al. (2019) [25]	18.4%	39.3%	44.4%	75%
Serapinas et al. (2020) [18]	0 cases^†^	0 cases	0 cases	100%
Lu et al. (2020) [20]	Overall: 33.3%			
Luo et al. (2020) [21]	Overall: 34.9%			
Lu et al. (2021) [26]	12.5%	66.7%	51.7%	83.3%
Lüthgens et al. (2021) [27]	29%	57.5%	29.7%	80%
Luo et al. (2021) [28]	23.1%	50%	36%	27.3%

^†^One case was screened by cffDNA test and was miscarried before invasive test.

**Table 3:** Prevalence of a positive cffDNA test result for autosomal trisomies.

	**T21**	**T13**	**T18**
Taneja et al. (2017) [15]	1.9%	0.3%	0.6%
Liang et al. (2018) [16]	0.5%	0.1%	0.2%
Lee et al. (2019) [17]	5.5%	0.9%	3.7%
Serapinas et al. (2020) [18]	1.2%	0 cases	0.4%
Wan et al. (2020) [19]	0.3%	0.1%	0.1%
Lu et al. (2020) [20]	0.4%	0.1%	0.1%
Luo et al. (2020) [21]	0.4%	0.1%	0.1%
Alyafee et al. (2021) [22]	3.5%	0.5%	2.5%

Abbreviations: PPV: positive predictive value; T13: trisomy 13; T18: trisomy 18; T21: trisomy 21.

**Table 4:** Prevalence of a positive cffDNA test result for SCAs.

	**45,X0**	**47,XXY**	**47,XXX**	**47,XYY**
Zhang et al. (2017) [23]	0.3%	0.1%	0.1%	0.03%
Liang et al. (2018) [16]	Overall: 0.3%^‡^			
Ma et al. (2018) [24]	0.2%	0.1%	0.1%	0.1%
Deng et al. (2019) [25]	0.3%	0.2%	0.1%	0.03%
Serapinas et al. (2020) [18]	0.1%^†^	0 cases	0 cases	0.1%
Lu et al. (2020) [20]	Overall: 0.4%^‡^			
Luo et al. (2020) [21]	Overall: 0.6%^‡^			
Lu et al. (2021) [26]	0.3%	0.2%	0.1%	0.1%
Lüthgens et al. (2021) [27]	Overall: 0.5%			
Luo et al. (2021) [28]	0.2%	0.2%	0.2%	0.1%

^†^One case was screened by cffDNA test and was miscarried before invasive testing.

^‡^Aggregate data across all SCAs was the only information provided by the authors.

## Data Availability

Data sharing is not applicable to this article as no datasets were generated or analyzed. The data that support the findings of this review are derived from the studies included in this review.

## Supporting Information

Supporting Information 1 Figure S1. PRISMA flow diagram: from initial search to final selection of articles.

Supporting Information 2 Table S1. Detailed characteristics of the included studies.

Supporting Information 3 Table S2. Results of the analysis of risk of bias in cohort studies using the CASP checklist.

Supporting Information 4 Appendix S1. The search strings employed for the study, by database.

Supporting Information 5 Appendix S2. Methodological quality of the included studies.
