# Supplementary material for: Cell-Free Fetal DNA for Prenatal Screening of Aneuploidies and Autosomal Trisomies: A Systematic Review
Source: Int J Pediatr. 2024 Oct 23;2024:3037937. doi: 10.1155/2024/3037937 (PMC11524709; doi:10.1155/2024/3037937)
Supplement: Supporting Information 1 — Figure S1. PRISMA flow diagram: from initial search to final selection of articles. [file 3037937.f1.docx]

***Figure S1.*** *PRISMA flow diagram: from initial search to final selection of articles. The stages of the selection process are indicated in the vertical boxes, on the left (PRISMA 2020).*
