# Supplementary material for: Cell-Free Fetal DNA for Prenatal Screening of Aneuploidies and Autosomal Trisomies: A Systematic Review
Source: Int J Pediatr. 2024 Oct 23;2024:3037937. doi: 10.1155/2024/3037937 (PMC11524709; doi:10.1155/2024/3037937)
Supplement: Supporting Information 2 — Table S1. Detailed characteristics of the included studies. [file 3037937.f2.zip › Table_S1_SuppInfo_ (1).docx]

**Table S1.** Detailed characteristics of the included studies.

| Author, year | Study type | Country | Study  duration | N^o^ of  participants | Participants | CffDNA sequencing technology | Diagnostic  confirmation |  |
| --- | --- | --- | --- | --- | --- | --- | --- | --- |
| Zhang B, 2017 | Retrospective | China | 4 years | 10 275 | MA: 18 to 50 years. GA: 13 to 27 GW. | MPS (whole genome) | Prenatal (amnio or cordocentesis) or postnatal karyotype. |  |
| Taneja PA, 2017 | Retrospective cohort | USA | 1.5 years | 5 579 | MA: 14 to 51 years. GA: ≥23 GW. | MPS (whole genome) | Karyotype. |  |
| Liang D, 2018 | Retrospective cohort | China | 5.3 years | 32 431 | MA: average of 30. GA: 12 to 26 GW. | MPS (whole genome) | Prenatal karyotype and/or CMA (amnio). |  |
| Ma L, 2018 | Retrospective cohort | China | 2.6 years | 6 002 | MA: unknown. GA: 13 to 22 GW. | Semiconductor sequencing platform | Prenatal karyotype. |  |
| Deng C, 2019 | Retrospective observational | China | 2.3 years | 50 301 | MA: 18 to 50 years. GA: 13 to 27 GW. | MPS (whole genome) | Prenatal karyotype (amnio or cordocentesis). |  |
| Lee DE, 2019 | Retrospective nested case-control | South  Korea | NA | 1 055 | MA: 22 to 48 years. GA: 10 to 21.4 GW. | MPS (shotgun) | Prenatal karyotype and QF-PCR (CVS or amnio). |  |
| Serapinas D, 2020 | Retrospective cohort | Lithuania | 6 years | 862 | MA: 19 to 40 years. GA: 9 to 21 GW. | SNP-based | Karyotype (CVS or amnio), or fetal liver biopsy after miscarriage. |  |
| Wan JH, 2020 | Retrospective cohort | China | 3 years | 1 539 | MA: unknown.  GA: ≥23 GW. | MPS (whole genome) | Prenatal (amnio) or postnatal karyotype, or postnatal placental investigation. |  |
| Lu W, 2020 | Retrospective | China | 3 years | 37 006 | MA: 18 to 54 years. GA: 12 to 32 GW. | Combinatorial probe-anchor synthesis | Prenatal (amnio) or postnatal karyotype. |  |
| Luo Y, 2020 | Retrospective cohort | China | 8 years | 40 311 | MA: 19 to 44 years. GA: 12 to 36 GW. | MPS (whole genome) | Prenatal karyotype + FISH (CVS, amnio, or cordocentesis). |  |
| Lu X, 2021 | Retrospective | China | 4 years | 45 773 | MA: 16 to 45 years. GA: 12 to 26 GW. | MPS (whole genome) | Prenatal karyotype (amnio). |  |
| Lüthgens K, 2021 | Retrospective cohort | Germa-ny | 1.6 years | 93 048 | MA: 31.1-37.5 years (IQR).  GA: 11.4-13.4 (IQR). | Microarray quantification of DANSR (Digital Analysis of Selected Regions) assays | Prenatal (amnio or CVS) or postnatal karyotype. |  |
| Luo Y, 2021 | Retrospective cohort | China | 6.5 years | 34 717 | MA: 18 to 50 years. GA: 12 to 36 GW. | MPS (shotgun) | Prenatal fetal karyotype and FISH (CVS, amnio, or cordocentesis), or postnatal karyotype. |  |
| Alyafee Y, 2021 | Prospective | Saudi Arabia | 0.6 years | 200 | MA: 21 to 48 years. GA: 10 to 32 GW. | MPS (whole genome) | Prenatal karyotype and FISH (CVS or amnio) |  |
| MA: maternal age; GA: gestational age; GW: gestational weeks; MPS: massive parallel sequencing; SNP: single nucleotide polymorphisms; CMA: chromosomal microarray analysis; QF-PCR: quantitative fluorescence polymerase chain reaction; Amnio: amniocentesis; CVS: chorionic villus sampling; FISH: fluorescence in situ hybridization; IQR: interquartile range. | | | | | | | | |
