# Supplementary material for: Cell-Free Fetal DNA for Prenatal Screening of Aneuploidies and Autosomal Trisomies: A Systematic Review
Source: Int J Pediatr. 2024 Oct 23;2024:3037937. doi: 10.1155/2024/3037937 (PMC11524709; doi:10.1155/2024/3037937)
Supplement: Supporting Information 3 — Table S2. Results of the analysis of risk of bias in cohort studies using the CASP checklist. [file 3037937.f3.docx]

**Table S2.** Results of the analysis of risk of bias in cohort studies using the CASP checklist

| Study | 1. Did the study address a clearly focused issue? | 2. Was the cohort recruited in an acceptable way? | 3. Was the exposure accurately measured to minimise bias? | 4. Was the outcome accurately measured to minimise bias? | 5a. Have the authors identified all important confounding factors? | 5b. Have they taken account of the confounding factors? | 6a. Was the follow up of subjects complete enough? | 6b. Was the follow up of subjects long enough? |
| --- | --- | --- | --- | --- | --- | --- | --- | --- |
| Zhang B (2017) | Yes | Yes | Yes | Yes | Yes | Yes | No | No |
| Taneja PA (2017) | Yes | Yes | Yes | Yes | Yes | Yes | No | Yes |
| Liang D (2018) | Yes | Yes | Yes | Yes | Yes | Yes | No | No |
| Ma L (2018) | Yes | Yes | Yes | Yes | Yes | Yes | No | No |
| Deng C (2019) | Yes | Yes | Yes | Yes | Yes | Yes | No | No |
| Serapinas D (2020) | Yes | Yes | Yes | Yes | Yes | Yes | No | No |
| Wan JH (2020) | Yes | Yes | Yes | Yes | Yes | Yes | No | Yes |
| Lu W (2020) | Yes | Yes | Yes | Yes | Yes | Yes | No | No |
| Luo Y (2020) | Yes | Yes | Yes | Yes | Yes | Yes | No | No |
| Lu X (2021) | Yes | Yes | Yes | Yes | Yes | Yes | No | No |
| Lüthgens K (2021) | Yes | Yes | Yes | Yes | Yes | Yes | No | No |
| Luo Y (2021) | Yes | Yes | Yes | Yes | Yes | Yes | No | No |
| Alyafee Y (2021) | Yes | Yes | Yes | Yes | Yes | Yes | Yes | Yes |

**Table S2.** (continued)

| 7. What are the results of this study? | 8. How precise are the results? | 9. Do you believe the results? | 10. Can the results be applied to the local population? | 11. Do the results of this study fit with other available evidence? | 12. What are the implications of this study for practice? |
| --- | --- | --- | --- | --- | --- |
| NIPT is useful for SCA screening, but the accuracy needs to be improved, especially for Monosomy X. | PPV for each individual aneuploidy. | Yes | Yes | Yes | Findings are consistent with current clinical practice recommendations but are not robust enough to make modifications. |
| NIPT is accurate for autosomal aneuploidy screening. | PPV for each individual aneuploidy. | Yes | Can't tell | Yes |  |
| NIPT is more reliable for T21 and T18, than for T13 screening. It is also accurate for SCAs. | PPV for each autosomal aneuploidy, but only overall PPV for SCAs. | Yes | Yes | Yes |  |
| SCAs screening is feasible with NIPT. | Only Overall PPV. | Yes | No | Yes |  |
| NIPT is useful to detect SCAs, but the accuracy needs to be improved, especially for Monosomy X. | PPV for each individual aneuploidy. | Yes | Yes | Yes |  |
| NIPT is a reliable screening tool but should not substitute first trimester screening. | PPV for each individual aneuploidy. | Yes | Yes | Yes |  |
| NIPT is accurate for autosomal aneuploidy screening. | Only overall PPV. | Yes | No | Yes |  |
| NIPT is more accurate for T21, than for T18, T13 and SCAs screening. | PPV for T21, T18, T13, but only overall PPV for SCAs. | Yes | Yes | Yes |  |
| NIPT yields high PPVs, except for T13 and SCAs. | PPV for T21, T18, T13, but only overall PPV for SCAs. | Yes | Yes | Yes |  |
| NIPT detects better sex chromosome trisomies than 45,X0. | PPV for each individual aneuploidy. | Yes | Yes | Yes |  |
| NIPT can detect SCAs, although PPVs are low. PPV depends on diagnostic test type, especially for 45,X0. | PPV for each individual aneuploidy. | Yes | Can't tell | Can’t tell |  |
| NIPT detects better sex chromosome trisomies, than 45,X0. | PPV for each individual aneuploidy. | Yes | Yes | Yes |  |
| High accuracy of NIPT to detect autosomal trisomies. | PPV for each individual aneuploidy. | Yes | Can't tell | Yes |  |
