# Supplementary material for: Cell-Free Fetal DNA for Prenatal Screening of Aneuploidies and Autosomal Trisomies: A Systematic Review
Source: Int J Pediatr. 2024 Oct 23;2024:3037937. doi: 10.1155/2024/3037937 (PMC11524709; doi:10.1155/2024/3037937)
Supplement: Supporting Information 5 — Appendix S2. Methodological quality of the included studies. [file 3037937.f5.docx]

Appendix S2. Methodological quality of the included studies.

The CASP cohort study and case-control study checklists were used to assess the risk of bias depending on the study design. Given that 13 of the 14 included studies consist of cohort studies, the CASP checklist for cohort studies was used to assess the risk of bias, and a summary of the results is presented in *Table S2.* Overall, the majority of studies can be classified as low risk of bias, although there were concerns regarding poor long-term follow-up, especially for the studies on SCAs. It is important to note that the results of 8 of the 13 cohort studies could be extrapolated and would be applicable to the local obstetric population because they were performed in an unselected population.^18,19,22-25,27,28^ Meanwhile, the results of the remaining 5 cohort studies may not be applicable to the local population for the following reasons: 2 studies only recruited participants with a high-risk pregnancy,^16,21^ 2 other studies did not define the risk level of the cohort,^17,26^ and a single study had a very low cohort size.^29^

As for Lee DE *et al.*’s 2019 study, it is the only nested case-control study of this review so the CASP checklist for case-control studies consisting of 11 questions was used for this particular study and yielded a low-risk of bias result.^20^ However, as it is a case-control study, it is intrinsically more prone to bias than the other 13 cohort studies.
