# Supplementary material for: Cell-Free Fetal DNA for Prenatal Screening of Aneuploidies and Autosomal Trisomies: A Systematic Review
Source: Int J Pediatr. 2024 Oct 23;2024:3037937. doi: 10.1155/2024/3037937 (PMC11524709; doi:10.1155/2024/3037937)
Supplement: Supplementary file 8 [file 3037937.f4.zip › 3037937.f4.odt]

Appendix S1. The search strings employed for the study, by database. 

| Database | Search string |
| --- | --- |
| PubMed | (((cell-free fetal DNA[MeSH Terms]) OR (cfdna[MeSH Terms]) OR (cell free fetal dna[MeSH Terms]) OR (circulating dna[MeSH Terms]) OR (cell free dna[MeSH Terms]) OR (non?invasive prenatal test*[MeSH Terms])) AND ((aneuploid*[MeSH Terms]) OR (trisom*[MeSH Terms]) OR (chromosom* anomal*[MeSH Terms]) OR (sex chromosom* aneuploid*[MeSH Terms]) OR (sex chromosom* anomal*[MeSH Terms])) AND (y_5[Filter])) AND (((cohort) OR (RCT) OR (randomi?ed controlled trial) OR (case-control) OR (prospective) OR (retrospective) OR (cross-sectional) OR (observational) ) OR (clinical trial)) |
| Web of  Science | ((TS=(cell-free fetal DNA) OR TS=(cffdna) OR TS=(cfdna) OR TS=(cell free fetal dna) OR TS=(cfdna) OR TS=(circulating dna) OR TS=(non*invasive prenatal testing) OR TS=(cfdna) OR TS=(ffdna) OR TS=(NIPT)) AND (TS=(trisom*) OR TS=(aneuploid*) OR TS=(sex chromosom* aneuploid*) OR TS=(sex chromosom* anomal*)) AND (TS=(RCT) OR TS=(randomized controlled trial) OR TS=(cohort) OR TS=(prospective) OR TS=(retrosective) OR TS=(case-control) OR TS=(cross-sectional) OR TS=(observational))) |
| Scopus | (TITLE-ABS-KEY(cell-free fetal DNA) OR TITLE-ABS-KEY(cffdna) OR TITLE-ABS-KEY(cfdna) OR TITLE-ABS-KEY(cell free fetal dna) OR TITLE-ABS-KEY(cfdna) OR TITLE-ABS-KEY(circulating dna) OR TITLE-ABS-KEY(non*invasive prenatal testing) OR TITLE-ABS-KEY(cfdna) OR TITLE-ABS-KEY(ffdna) OR TITLE-ABS-KEY(NIPT)) AND (TITLE-ABS-KEY(trisom*) OR TITLE-ABS-KEY(aneuploid*) OR TITLE-ABS-KEY(sex chromosom* aneuploid*) OR TITLE-ABS-KEY(sex chromosom* anomal*)) AND (TITLE-ABS-KEY(RCT) OR TITLE-ABS-KEY(randomized controlled trial) OR TITLE-ABS-KEY(cohort) OR TITLE-ABS-KEY(Prospective) OR TITLE-ABS-KEY(retrospective) OR TITLE-ABS-KEY(clinical trial) OR TITLE-ABS-KEY(case-control) OR TITLE-ABS-KEY(cross-sectional) OR TITLE-ABS-KEY(obervational)) |
